# Supplementary material for: How much synthetic oxytocin is infused during labour? A review and analysis of regimens used in 12 countries
Source: PLoS One. 2020 Jul 28;15(7):e0227941. doi: 10.1371/journal.pone.0227941 (PMC7386656; doi:10.1371/journal.pone.0227941)
Supplement: Attachment — Submitted filename: Response to reviewers.docx [file pone.0227941.s004.docx]

| Manuscript: How much synthetic oxytocin is infused during labour? A review and analysis of regimens used in 12 countries  [PONE-D-19-21810] | |
| --- | --- |
| Editor’s comments | Authors’ response to reviewers’ comments |
| 1. Please include additional information regarding the survey or questionnaire used in the study and ensure that you have provided sufficient details that others could replicate the analyses. For instance, please give more details on how the survey was distributed, hoe many participants per each country were invited (and the inclusion and exclusion criteria used), how participating countries were chosen.  Moreover, if you developed a questionnaire as part of this study and it is not under a copyright more restrictive than CC-BY, please include a copy, in both the original language and English, as Supporting Information.  Moreover, please include more details on how the questionnaire was pre-tested, and whether it was validated. | We have included the following text [p7, lines 131-143]:  The study was conducted as part of the COST Action IS1405 (BIRTH) (2014-2018) ([https://www.cost.eu/actions/IS1405/BIRTH](https://www.cost.eu/actions/IS1405/#tabs\|Name:overview)), a network which aimed to advance scientific knowledge of the normal physiology of labour and birth.  An email seeking expressions of interest in the review was sent to the 51 registered members of the Action’s working group 4, based in 20 countries. To take part in the review, members needed knowledge of their country’s/region’s/local hospital’s maternity services guideline on use of intrapartum oxytocin on induction and acceleration of labour. There were no exclusion criteria. A total of 18 members from 12 countries expressed an interest in taking part in the review.  A core group developed a data collection form based on the criteria used in the Irish clinical practice guideline on oxytocin to induce or accelerate labour.^4^ The form was then sent to the 18 participants for content validation and review. Subsequently, one item was included on the persons responsible for developing the guideline (obstetricians, midwives, users) and the final form contained 24 items (S1).  The data collection form has been included as Supporting Information (S1). |
| 2. Please provide additional details regarding participant consent. In the ethics statement in the Methods and online submission information, please ensure that you have specified (1) whether consent was informed and (2) what type you obtained (for instance, written or verbal). If your study included minors, state whether you obtained consent from parents or guardians. If the need for consent was waived by the ethics committee, please include this information. | We have included the following text [p8, lines 158-164]:  Ethics  As the study was a review of available national, regional and/or local regimens on intrapartum oxytocin administration, ethics committee approval was not required. The data collection form was anonymous, and information on the country and whether the guideline was national, regional or hospital-level was required. Hospital-level guidelines were identified as ‘Hospital 1, 2 etc.’ but not by name. No participant or individual-level data were collected.  Written consent was not required. Participants volunteered to provide information requested. |
| Reviewer #1’s comments |  |
| Points 1-4: | No comments required |
| Point 5.  Reviewer #1: Interesting and well-designed study. Needs minor editing for compound sentences and minor grammatical errors, such as changing "exceeded" to "exceeds" from lines 188-193. | Thank you, we have corrected the errors and edited the paper. |
| Tables are very detailed, but I think Table 3 is actually too much detail; consider changing this to brief paragraph summaries of different countries' criteria.  Similarly- The second part of Table 5, demonstrating all of the possible dosing per each algorithm, detracts from the impact of the work rather than adding to it. I realize this table is the bulk of the outcome of your carefully crafted study, but it is unlikely that a reader would carefully analyze the math. Perhaps including one or two country's data, with an appendix or link out to the full data set? The example at line 404 is terrific and could be bolstered by adding a contrasting example. The big picture differences in initial dose, escalation protocols, and total dose are the staggering outcomes, and you have laid these out well without needing such detail. | We have removed table 3 and included it as supporting information, S2.  We have removed table 5 and included it as supplemental information, S3. We have inserted Germany’s minimum and maximum regimens as an example of the lowest and highest regimens, and inserted a brief summary of other regimens (Table 4). |
| The multitude of recent studies and meta-analysis regarding induction at 39 weeks and improved maternal and neonatal outcomes would argue against your tenet of over-medicalizing labor, but looking at the association of bad outcomes with possible injudicious use of exogenous oxytocin remains very important- did you look at any institutional data on cesarean rate, uterine rupture rate, or any other discrete poor outcomes of the differing protocols? Clearly could be a robust additional study but would entice the reader to think about the implications of these differences | We did not explore maternal and neonatal outcomes alongside any of these regimens but acknowledge that it would be an interesting and very valuable study. We hope others will build on this work and explore outcomes alongside the regimens.    We did not explore outcomes associated with induction of labour because there are different practices across European countries and within countries. For example, Denmark induces women at 41^+3-4^ weeks’ gestation; Sweden is discussing inducing women at 41 weeks rather than at 42 weeks’ gestation, and the Norwegian guidelines states that an ultrasound and CTG should be performed at week 41^+0-3^ with close follow-up and induction no later than week 42^+0.^  Our central argument is that there is considerable variation in the total dose of infused oxytocin between regimens and that these variations represent ‘over’ use of infused oxytocin in some settings, if lower doses are effective. |
